# Case report: fulminant myocarditis with biopsy-proven eosinophilic infiltration and burned-out myocardial pathology at autopsy

**DOI:** 10.1093/ehjcr/ytag562

**Published:** 2026-07-24

**Authors:** Rui Katano, Atsushi Tada, Hirokazu Sugino, Toshiyuki Nagai, Toshihisa Anzai

**Affiliations:** Department of Cardiovascular Medicine, Faculty of Medicine and Graduate School of Medicine, Hokkaido University, Kita 15, Nishi 7, Kita-ku, Hokkaido 060-8638, Sapporo, Japan; Department of Cardiovascular Medicine, Faculty of Medicine and Graduate School of Medicine, Hokkaido University, Kita 15, Nishi 7, Kita-ku, Hokkaido 060-8638, Sapporo, Japan; Department of Cancer Pathology, Faculty of Medicine and Graduate School of Medicine, Hokkaido University, Kita 15, Nishi 7, Kita-ku, Hokkaido 060-8638, Sapporo, Japan; Department of Cardiovascular Medicine, Faculty of Medicine and Graduate School of Medicine, Hokkaido University, Kita 15, Nishi 7, Kita-ku, Hokkaido 060-8638, Sapporo, Japan; Department of Cardiovascular Medicine, Faculty of Medicine and Graduate School of Medicine, Hokkaido University, Kita 15, Nishi 7, Kita-ku, Hokkaido 060-8638, Sapporo, Japan

**Keywords:** Myocarditis, Biopsy, Autopsy, Mechanical circulatory support, Immunosuppressive therapy, Case report

## Abstract

**Background:**

Fulminant myocarditis causes severe haemodynamic instability, requiring mechanical or inotropic support. In this setting, early endomyocardial biopsy is crucial to identify specific subtypes like eosinophilic or giant cell myocarditis, which require immediate and targeted immunosuppressive intervention.

**Case summary:**

A 54-year-old female patient presented to a local clinic with several days of fever and dyspnoea, presenting in a state of shock with markedly elevated cardiac enzymes. Although electrocardiography and transthoracic echocardiography findings initially suggested acute myocardial infarction, coronary angiography revealed no significant stenosis. Consequently, fulminant myocarditis was suspected. Following cardiac arrest and initiation of venoarterial extracorporeal membrane oxygenation and Impella CP, she was transferred to our hospital. Endomyocardial biopsy revealed myocardial necrosis with inflammatory cell infiltration, including prominent degranulating eosinophils, consistent with eosinophilic myocarditis. Despite steroid pulse therapy and escalation of immunosuppression with tacrolimus and mycophenolate mofetil, the patient developed persistent electrical silence without mechanical recovery and died. Autopsy revealed near-complete loss of viable cardiomyocytes with chronic inflammation and interstitial fibrosis, consistent with burned-out myocardial pathology.

**Discussion:**

This case highlights the devastating nature of fulminant myocarditis, specifically when characterized by extensive and irreversible cardiomyocyte destruction. Once massive myocardial loss has occurred, conventional interventions—including mechanical circulatory support and intensive immunosuppressive therapy—may reach their therapeutic limits. This clinical-pathological correlation underscores the critical necessity of early risk stratification to identify patients at risk for profound tissue damage. Moving forward, refining strategies for timely therapeutic escalation and establishing realistic clinical goals remain pivotal challenges in the management of this lethal pathophysiology.

Learning points

- Once massive cardiomyocyte loss has occurred, recovery may be limited despite advanced mechanical circulatory support and immunosuppressive therapy.
- Early recognition of patients at risk for irreversible myocardial destruction remains essential.

## Introduction

Fulminant myocarditis is an acute myocarditis characterized by severe haemodynamic compromise requiring inotropes and/or mechanical circulatory support (MCS).^1^ Among the spectrum of myocarditis, establishing a histological diagnosis of eosinophilic or giant cell myocarditis by endomyocardial biopsy (EMB) is particularly important, as these subtypes have critical implications for the timely initiation of immunosuppressive therapy.^2^ We report a fatal case of fulminant myocarditis progressing to burned-out myocardial pathology despite intensive supportive care.

## Summary figure

**Figure:**
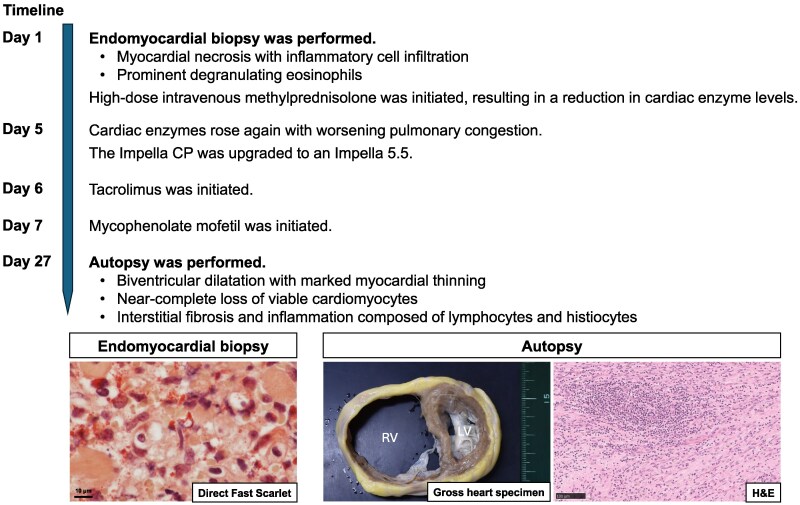


## Case presentation

The case was a 54-year-old woman with a history of hypertension and dyslipidemia who presented to a local clinic with several days of fever and dyspnoea. She was hypotensive, with markedly elevated cardiac enzymes. Electrocardiography (ECG) showed ST-segment elevation in leads I, aVL, and V4–6. Transthoracic echocardiography (TTE) revealed regional left ventricular (LV) systolic dysfunction involving the lateral and posterior wall with pericardial effusion. She subsequently experienced cardiac arrest and was transferred to another hospital on mechanical ventilation and venoarterial extracorporeal membrane oxygenation (VA-ECMO). Although acute myocardial infarction was initially suspected, the absence of mechanical complications and significant coronary stenosis on angiography made this diagnosis unlikely, raising suspicion for fulminant myocarditis. After Impella CP insertion, she was transferred to our hospital.

Upon arrival, her blood pressure was 77/74 mmHg, heart rate 77 beats/min, and body temperature 36.8°C. She was supported with VA-ECMO at 2.9 L/min and an Impella CP providing 1.7 L/min at performance level P3, without catecholamine. Laboratory testing demonstrated markedly elevated cardiac biomarkers, including creatine kinase of 1518 U/L, troponin T of 33 790 ng/L, and N-terminal prohormone of brain natriuretic peptide of 24 624 pg/mL. The peripheral eosinophil count was <500 cells/mm^3^, and there was no history of recent drug use or vaccination. Additionally, tests for antinuclear and antineutrophil cytoplasmic antibodies were negative. Computed tomography and chest radiography revealed bilateral pulmonary infiltration (*Figure 1A* and *B*), and ECG showed sinus rhythm, low voltage, and a wide QRS complex (*Figure 2A*). TTE showed global LV akinesis, severe right ventricular (RV) hypokinesis, circumferential LV wall thickening with marked cavity reduction, and minimal pericardial effusion (see Supplementary material online, *Video S1*). EMB obtained from the RV revealed myocardial necrosis with inflammatory cell infiltration, including prominent degranulating eosinophils (*Figure 3A–C*), consistent with eosinophilic myocarditis (EM).

**Figure 1:**
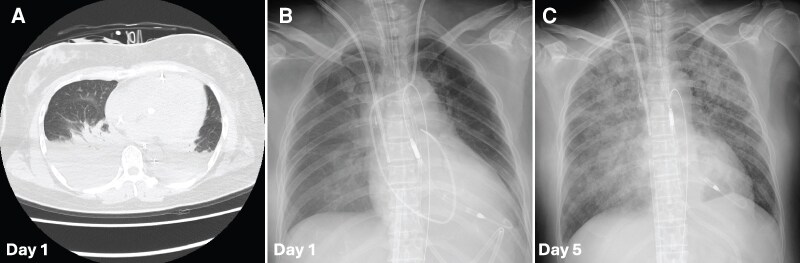
**(** *A* **)** Day 1: chest computed tomography showing bilateral pulmonary infiltration. **(***B***)** Day 1: chest radiography showing pulmonary oedema. **(***C***)** Day 5: chest radiography showing pulmonary congestion.

**Figure 2:**
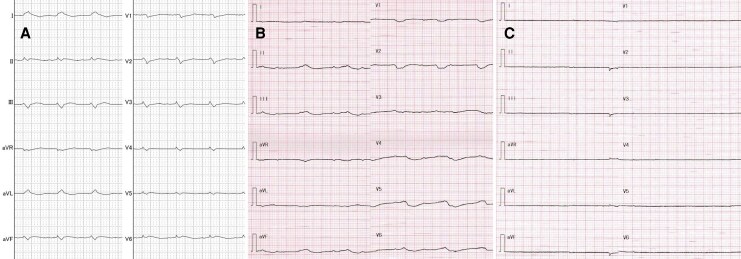
Serial electrocardiogram findings. **(***A***)** Day 1: electrocardiogram showing sinus rhythm, low voltage in all leads, and wide QRS. **(***B***)** Day 4: electrocardiogram showing a slight increase in R-wave amplitude. **(***C***)** Day 10: electrocardiogram showing asystole.

**Figure 3:**
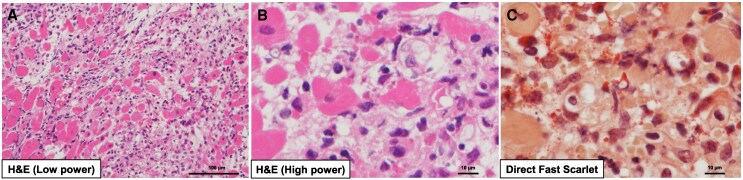
Right ventricular endomyocardial biopsy specimens. Haematoxylin and eosin staining (*A*, *B*) and direct fast scarlet (*C*) staining demonstrating myocardial necrosis with inflammatory cell infiltration, including prominent degranulating eosinophils. Scale bars: 100 μm (*A*), 10 μm (*B*, *C*). H&E, haematoxylin and eosin; DFS, direct fast scarlet.

In accordance with the guidelines,^3^ high-dose intravenous methylprednisolone (1000 mg/day for 3 days) was initiated, resulting in an initial reduction in cardiac enzyme levels (*Figure 4*). Because the LV cavity was nearly obliterated, leading to suction events, the Impella performance level was reduced to P1. On Day 4, ECG showed a slight increase in R-wave amplitude (*Figure 2B*), and TTE demonstrated modest improvement in LV wall motion and wall thickness (see Supplementary material online, *Video S2*), prompting transition to maintenance prednisolone (1.0 mg/kg/day).

**Figure 4:**
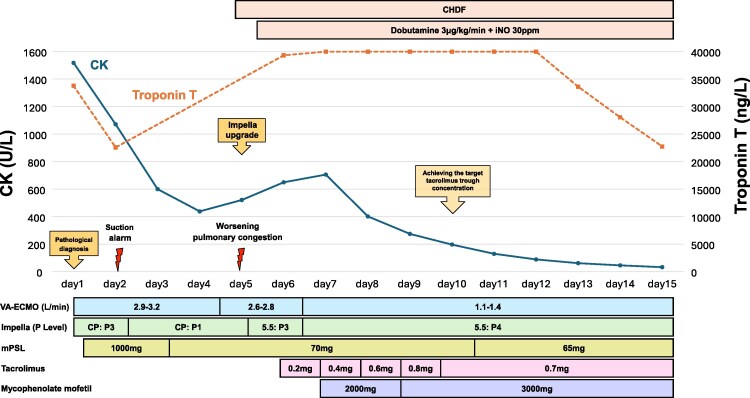
Clinical course and therapeutic timeline. The upper panel shows serial changes in the creatine kinase (CK; solid line) and troponin T (dashed line). Troponin T levels exceeded the upper limit of quantification (>40 000 ng/L) from Day 7 to Day 12. The lower panel illustrates the status of mechanical circulatory support and the administration of immunosuppressive therapy. CK, creatine kinase; mPSL, methylprednisolone; PSL, prednisolone; VA-ECMO, venoarterial extracorporeal membrane oxygenation; CHDF, continuous haemodiafiltration; iNO, inhaled nitric oxide.

From Day 5 onward, cardiac enzymes rose again with worsening pulmonary congestion (*Figure 1C* and *4*), and continuous haemodiafiltration was initiated for acute kidney injury. The Impella CP was upgraded to an Impella 5.5 due to frequent suction events caused by septal interference and the device-related moderate aortic regurgitation, which limited effective LV unloading. Despite adjunctive therapy with dobutamine (3.0 μg/kg/min) and inhaled nitric oxide to facilitate left ventricular unloading, the haemodynamic response was inadequate, and the Impella 5.5 achieved <1.0 L/min of flow at performance level P3.

Given the rapidly progressive course and lack of recovery despite intensive support for EM, a more aggressive immunosuppressive strategy was pursued for suspected severe immune-mediated myocarditis. Accordingly, tacrolimus was initiated on Day 6 with a target trough concentration of 10–15 ng/mL, and mycophenolate mofetil was added on Day 7. Cardiac enzyme levels began to decline from Day 7, and the Impella 5.5 flow increased to ∼1.5 L/min at performance level P4. However, despite achieving the target tacrolimus trough concentration by Day 10, there was no recovery of electrical or mechanical cardiac activity, and ECG and TTE continued to demonstrate an asystolic state (*Figure 2C*; see Supplementary material online, *Video S3*). Advanced therapies, including durable mechanical support as a bridge to transplantation, were also considered. However, profound biventricular failure and progressive multiorgan dysfunction made meaningful recovery unlikely even with further escalation of support. Given the absence of myocardial recovery, the limited potential for cardiac recovery was explained to the patient’s family. Following careful discussion, a decision was made to discontinue MCS, and the patient died on Day 27.

Autopsy revealed a heart weight of 250 g, without cardiomegaly. Macroscopically, the heart showed biventricular dilatation with marked myocardial thinning (LV wall 10 mm, RV wall 1 mm) and a softened myocardial texture, without mural thrombi (*Figure 5A* and *B*). Microscopically, there was near-complete loss of viable cardiomyocytes, with interstitial fibrosis and inflammation composed of lymphocytes and histiocytes (*Figure 5C–F*). No eosinophils or multinucleated giant cells were identified. Owing to the advanced degree of myocardial degeneration, the sinoatrial and atrioventricular nodal cells were indistinct.

**Figure 5:**
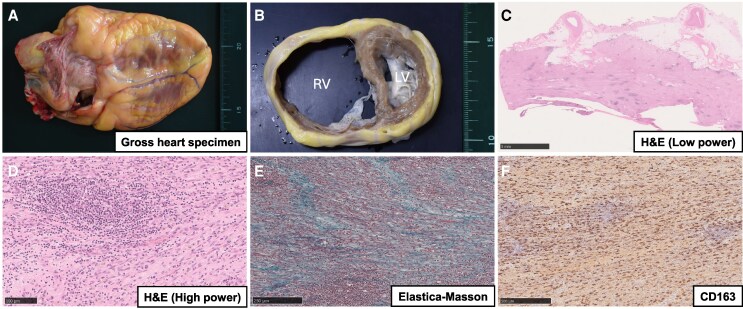
Autopsy findings. The heart weighed 250 g without evidence of cardiomegaly. (*A*, *B*) Gross examination showing biventricular dilatation with marked myocardial thinning (left ventricular wall thickness, 10 mm; right ventricular wall thickness, 1 mm) and a softened texture, without mural thrombi. (*C–F*) Histological sections from the anterior wall of the left ventricle stained with haematoxylin and eosin (*C*, *D*) and Elastica–Masson (*E*) and CD163 (*F*) revealing near-complete loss of viable cardiomyocytes with chronic inflammation, interstitial fibrosis, and chronic inflammatory cell infiltration predominantly composed of lymphocytes and histiocytes, notably in the absence of eosinophils or multinucleated giant cells. Scale bars: 5 μm (*C*), 100 μm (*D*), 250 μm (*E*), and 500 μm (*F*). RV, right ventricle; LV, left ventricle; H&E, haematoxylin and eosin.

## Discussion

We report a fatal case of fulminant myocarditis managed with MCS and immunosuppressive therapy. There is a case report of a patient with fulminant myocarditis who survived after 56 h of cardiac arrest supported by VA-ECMO.^4^ In the current case, despite intensive supportive and disease-directed treatment, the patient developed persistent electrical silence and ultimately died. Autopsy revealed extensive loss of cardiomyocytes and widespread replacement fibrosis, indicating that irreversible myocardial destruction had already occurred and corresponded closely with the clinical course. The need for MCS other than Impella, QRS duration >120 ms on initial ECG, and biventricular dysfunction are considered predictors of poor prognosis in myocarditis,^5^ all of which were present in the current case.

Mechanical unloading with Impella reduces LV wall stress and may attenuate inflammatory responses and myofibroblast activation.^6^ The PROPELLA concept has therefore been proposed to promote myocardial recovery through prolonged unloading in fulminant myocarditis. However, in the present case, frequent suction events, septal interference, and device-related aortic regurgitation limited effective unloading despite escalation to Impella 5.5. Although a transient decline in cardiac biomarkers was observed, this did not translate into electrical or mechanical recovery, suggesting that extensive myocardial loss had already progressed beyond a reversible stage.

Although eosinophilic infiltration was observed in myocardial tissue, the clinical course was unusually severe and atypical for EM, with rapidly progressive biventricular failure, refractory haemodynamics, and persistent asystole, and giant cell myocarditis was considered in the differential diagnosis. Importantly, giant cells were absent in the biopsy specimen; however, sampling error remains a significant limitation, with a reported sensitivity of 80%–93% within 2–4 weeks of disease onset.^7^ A significant diagnostic challenge lies in the fact that 24.1% of histologically confirmed cases do not present with peripheral eosinophilia, potentially leading to the underdiagnosis of this condition without an endomyocardial biopsy. Moreover, while EM is generally regarded as more responsive to corticosteroid therapy than giant cell myocarditis, several reports describe that EM is diagnosed only at autopsy, implying that the treatment responsiveness may be heterogeneous.^8^

This case highlights the lethal pathophysiology of fulminant myocarditis, characterized by massive myocardial loss, for which MCS and immunosuppressive therapy may be insufficient once irreversible damage has occurred. Earlier identification of patients at risk for extensive myocardial destruction and refinement of strategies for timely therapeutic escalation and goal setting remain important challenges for future investigation.

## Supplementary Material

ytag562_Supplementary_Data

## Data Availability

The data underlying this article are available in the article and in its online Supplementary material.

## Lead author biography

Dr Rui Katano is currently a postgraduate student in the Department of Cardiovascular Medicine at Hokkaido University Hospital. His clinical focus includes a wide range of cardiovascular diseases, with a particular interest in advanced heart failure, including heart transplantation. He completed his medical degree (MD) at Hokkaido University School of Medicine and finished his internal medicine residency at Sunagawa City General Hospital. Dr Katano is an active member of the Japanese Circulation Society and is dedicated to advancing the diagnosis, care, and treatment of complex cardiac conditions.

## Notes

**Consent:** Informed consent was obtained from the patient in accordance with the Committee on Publication Ethics (COPE) guidelines.

## References

[1] Ammirati E, Cipriani M, Lilliu M, Sormani P, Varrenti M, Raineri C, et al Survival and left ventricular function changes in fulminant versus nonfulminant acute myocarditis. Circulation 2017;136:529–545. 28576783 10.1161/CIRCULATIONAHA.117.026386

[2] Huang F, Ammirati E, Ponnaiah M, Montero S, Raimbault V, Abrams D, et al Fulminant myocarditis proven by early biopsy and outcomes. Eur Heart J 2023;44:5110–5124. 37941449 10.1093/eurheartj/ehad707

[3] Nagai T, Inomata T, Kohno T, Sato T, Tada A, Kubo T, et al JCS 2023 guideline on the diagnosis and treatment of myocarditis. Circ J 2023;87:674–754. 36908170 10.1253/circj.CJ-22-0696

[4] Sugamura K, Sugiyama S, Kawano H, Horio E, Ono S, Kojima S, et al Fulminant myocarditis survivor after 56 hours of non-responsive cardiac arrest successfully returned to normal life by cardiac resynchronization therapy: a case report. J Cardiol 2006;48:345–352. 17243629

[5] Schulz-Menger J, Collini V, Gröschel J, Adler Y, Brucato A, Christian V, et al 2025 ESC guidelines for the management of myocarditis and pericarditis. Eur Heart J 2025;46:3952–4041. 40878297 10.1093/eurheartj/ehaf192

[6] Spillmann F, Van Linthout S, Schmidt G, Klein O, Hamdani N, Mairinger T, et al Mode-of-action of the PROPELLA concept in fulminant myocarditis. Eur Heart J 2019;40:2164–2169. 30891599 10.1093/eurheartj/ehz124 PMC6612367

[7] Kandolin R, Lehtonen J, Salmenkivi K, Räisänen-Sokolowski A, Lommi J, Kupari M. Diagnosis, treatment, and outcome of giant-cell myocarditis in the era of combined immunosuppression. Circ Heart Fail 2013;6:15–22. 23149495 10.1161/CIRCHEARTFAILURE.112.969261

[8] Brambatti M, Matassini MV, Adler ED, Klingel K, Camici PG, Ammirati E. Eosinophilic myocarditis: characteristics, treatment, and outcomes. J Am Coll Cardiol 2017;70:2363–2375. 29096807 10.1016/j.jacc.2017.09.023

