# Supplementary material for: Case report: fulminant myocarditis with biopsy-proven eosinophilic infiltration and burned-out myocardial pathology at autopsy
Source: Eur Heart J Case Rep. 2026 Jul 24;10(8):ytag562. doi: 10.1093/ehjcr/ytag562 (PMC13431896; doi:10.1093/ehjcr/ytag562)
Supplement: ytag562_Supplementary_Data [file ytag562_supplementary_data.zip › Video Legends.docx]

**Video Legends**

Serial transthoracic echocardiographic findings. **(Video S1)** Day 1: Baseline echocardiography demonstrating global left ventricular akinesis, diffuse severe right ventricular hypokinesis, circumferential left ventricular wall thickening with marked cavity reduction, and minimal pericardial effusion. **(Video S2)** Day 4: Follow-up echocardiography showing modest improvement in left ventricular wall motion and wall thickness. **(Video S3)** Day 10: Subsequent echocardiography demonstrating no further recovery, with persistent electrical silence and an asystolic state.
